# Society Meetings

**Published:** 1905-10

**Authors:** 


### SOCIETY MEETINGS.

---

The thirty-seventh annual meeting of the Medical Association of Central New York will be held at Buffalo, Tuesday, October 24, 1905. The meeting—morning and afternoon sessions—will be held in the lecture room of the Buffalo Historical Society, Delaware Park. The morning session will be called to order at 10 o'clock; the afternoon session at 2.30 o'clock. The officers for 1905 are: president, Charles G. Stockton, Buffalo; vice-president, D. M. Totman, Syracuse; secretary, C. A. Greenleaf, Rochester; treasurer, Wm. M. Brown, Rochester.

The physicians of Buffalo and Western New York are cordially invited to attend and participate in the meeting.

---

The American Academy of Medicine will hold its thirtieth annual meeting in the Northwestern University building, corner Lake and Dearborn streets, Chicago, on Thursday and Friday, November 9 and 10, 1905.

---

The Homeopathic Medical Society of the State of New York held its semi-annual meeting at Syracuse, September 26 and 27, 1905, under the presidency of Dr. DeWitt G. Wilcox of Buffalo.

---

The Buffalo Academy of Medicine held two meetings during September. The first meeting of the academic year was held Tuesday, September 19, 1905, by the section on medicine. Program: The early diagnosis of tuberculosis, by John H. Pryor, of Sarnac Lake.

The second meeting was held September 26, by the section of obstetrics and gynecology. Program: Version versus high forceps in primiparæ, by Francis M. O'Gorman.

---

The New York and New England Association of Railway Surgeons will hold its fifteenth annual meeting at the Academy of Medicine, New York, November 17-18, 1905, under the presidency of Dr. G. P. Conn, of Concord, N. H. One half-day of the meeting will be devoted to a symposium on "Injuries to the Head and Spine." Noted surgeons will take part in the discussion. A cordial invitation is extended to the profession. The secretary is Dr. George Chaffee, of Brooklyn.

